# The effectiveness of culturally tailored video narratives on medication understanding and use self-efficacy among stroke patients: A randomized controlled trial study protocol

**DOI:** 10.1097/MD.0000000000010876

**Published:** 2018-06-01

**Authors:** Jamuna Rani Appalasamy, Kyi Kyi Tha, Kia Fatt Quek, Siva Seeta Ramaiah, Joyce Pauline Joseph, Anuar Zaini Md Zain

**Affiliations:** a Jeffrey Cheah School of Medicine and Health Sciences, Monash University Malaysia; b Subang Jaya Medical Centre, Jalan SS12/1a, Selangor; c Neurology Department, Hospital Kuala Lumpur, Kuala Lumpur, Malaysia.

**Keywords:** culturally tailored video narrative, Health Belief Model, Information-Motivation-Behavior model, medication understanding and use self-efficacy, patient education, randomized controlled trial, stroke

## Abstract

**Introduction:**

A substantial number of the world's population appears to end with moderate to severe long-term disability after stroke. Persistent uncontrolled stroke risk factor leads to unpredicted recurrent stroke event. The increasing prevalence of stroke across ages in Malaysia has led to the adaptation of medication therapy adherence clinic (MTAC) framework. The stroke care unit has limited patient education resources especially for patients with medication understanding and use self-efficacy. Nevertheless, only a handful of studies have probed into the effectiveness of video narrative at stroke care centers.

**Method:**

This is a behavioral randomized controlled trial of patient education intervention with video narratives for patients with stroke lacking medication understanding and use self-efficacy. The study will recruit up to 200 eligible stroke patients at the neurology tertiary outpatient clinic, whereby they will be requested to return for follow-up approximately 3 months once for up to 12 months. Consenting patients will be randomized to either standard patient education care or intervention with video narratives. The researchers will ensure control of potential confounding factors, as well as unbiased treatment review with prescribed medications only obtained onsite.

**Results:**

The primary analysis outcomes will reflect the variances in medication understanding and use self-efficacy scores, as well as the associated factors, such as retention of knowledge, belief and perception changes, whereas stroke risk factor control, for example, self-monitoring and quality of life, will be the secondary outcomes.

**Discussion and conclusion:**

The study should be able to determine if video narrative can induce a positive behavioral change towards stroke risk factor control via enhanced medication understanding and use self-efficacy. This intervention is innovative as it combines health belief, motivation, and role model concept to trigger self-efficacy in maintaining healthy behaviors and better disease management.

**Trial registration:**

ACTRN (12618000174280).

## 1 Introduction

Stroke has been an enormous burden of disease at the global scale with upper-middle-income countries recording the highest prevalence, followed by lower-middle-income and high-income nations, as reported by the global statistics in 2012.^[1,2]^ A substantial number of the population ends with moderate to severe long-term disability after stroke.^[3]^ Up to a quarter of people who experience a transient ischemic attack (TIA) or stroke will proceed to recurrent stroke within a few weeks or months.^[4]^ An alarming morbidity rate due to stroke could paralyze the economic growth caused by incurring treatment expenses and loss of workforce.^[5]^ Modifiable recurrent stroke risk factors (e.g., hypertension, diabetes, and hyperlipidemia) and non-modifiable risk factors, such as sex, age, and familial history, are added-on with barriers to adherence to medication, which could cause a substantial loss in terms of money, time, and effort of various stakeholders.^[6–9]^ Besides, a recent study associated adherence to recurrent stroke preventative medication with reduced stroke occurrence.^[10]^

An estimate of 40,000 acute stroke cases are recorded yearly in Malaysia, and about 23% of hospitalization cases referred to patients with a history of recurrent stroke.^[11]^ Approximately, ischemic incidence have been reported to increase annually by 29.5%, while almost 18.7% for hemorrhagic stroke.^[12]^ In total, 33% from acute strokes end with moderate to severe disability. Mortality and morbidity rates of stroke have remained high due to the aging of the population.^[11]^ Hence, stroke recurrence initiatives seem to focus on combatting modifiable risk factors adapted via behavioral interventions.^[13,14]^ While neurologists prescribe preventative stroke medications, a critical component of effective modifiable stroke risk factor control rests with the individual.

The increasing prevalence of stroke across ages in Malaysia have led to the adaptation of medication therapy adherence clinic (MTAC) framework to assist patient care after being discharged from outpatient clinics.^[15]^ Patient education, as a construct of MTAC, addresses modifiable individual behavioral factors identified during clinic appointments. However, limited resources are at grasp regarding the type of intervention that may enhance medication understanding and use self-efficacy so as to promote medication adherence, especially for diseases with various underlying comorbidities, such as stroke.

A highly activated individual takes the effort to acquire disease-specific knowledge and self-efficacy, which lead to engagement in positive health behavior.^[16–18]^ Self-determination theory developed by Deci and Ryan^[19,20]^ explains that without external influences, there is a link between human motivation and personality characteristics with basic needs of satisfaction among patients. They have classified self-motivation into; intrinsic and extrinsic motivation. Intrinsic motivation involves the person engaging in a behavior as he or she finds the activity enjoyable, whereas extrinsic motivation occurs when the person is motivated to modify behavior to earn a reward or to avoid negative consequences.^[19,21]^ Hence, if the person is highly motivated, adhering to a specific medication regime would be a simple routine, otherwise it would be a burden that can affect one's quality of life.

Information-Motivation-Behavior (IMB) is a notable health behavior change framework applicable to patient education intervention.^[22,23]^ Adaptation of IMB model in patient education delivered via information technology, audio-visual, and personalized counseling reported significant behavioral changes.^[24–26]^ The information provides knowledge about risk factors and types of behavior or barriers towards adherence. Motivation is comprised of personal attitude, belief, and perception towards activation of the individual response, while behavioral skills are specific learning skills that enable positive behavior modification. On the other hand, self-efficacy influences the patient's confidence towards the final execution of an action concerning healthy lifestyle and medication management.^[22,27]^

Within the context of IMB, the foundation of information developed using health belief model (HBM) constructs would deliver a system that enhances understanding and self-efficacy of the patient towards better medication management.^[28,29]^ HBM suggests that individuals protect their health depending on their belief of susceptibility to a disease condition, that the occurrence of their disease condition would have an impact on their quality of life. With that aim, they have options for actions to avoid their perceived disease condition. Additionally, HBM suggests that individuals consider benefits of taking the planned action which outweighs their costs.^[30,31]^ Therefore, patient education intervention should adapt behavioral theories and framework for further exploration of disease risk factor control.

With IMB and HBM to be adopted as the conceptual frameworks, the study is projected to evaluate the impact of patient education on medication understanding and use self-efficacy delivered via video narrative. Video narrative is a useful tool that provides knowledge, improves confidence, and promotes self-learning among patients with various diseases.^[32,33]^ Narratives, as personal stories from comrades or professionals, are seen as motivator, persuader or a role model for other patients to react to their behavior.^[34]^ It can also overcome resistance and facilitate information processing.^[35]^ Thus, a doctor's narrative would be a source of a genuine informant supplementing a patient's story that is believed to strengthen motivation towards behavioral changes so as to minimize the detrimental psychological effects of stroke.

Based on prior studies, stroke risk factors were sequenced in descending order starting with the most prevalent risk factor; hypertension, diabetes mellitus, hyperlipidemia, ischemic heart disease, and history of a previous stroke; thus demanding the need for focused efforts to reduce recurrent stroke prevalence.^[36,37]^ To the researchers’ knowledge, various studies have faced considerable challenges to motivate stroke patients due to their varied perspectives regarding stroke severity and medication management. Therefore, this study hypothesizes that narratives developed on behavioral constructs, framework, and experiences of doctors and patients possess the ability to generate substantial expected outcomes. Thus, the study will disclose the specific challenges of medication management among recurrent stroke patients for whom the planned intervention would be most beneficial.

## 2 Materials and methods

### 2.1 Study design and aim

This is a single-blind, randomized controlled, parallel group, longitudinal, and exploratory trial in which patients with risk of recurrent stroke will receive either a video narrative and standard care or standard care alone. This study is due to start in the month of May 2018. The study outcomes are assessed as follows; at baseline: T0, 3 months: T1, 6 months: T2, 9 months: T3, and 12 months: T4, as outpatient follow-up. The design and the conduct of the study conform to the revised Consolidated Standards of Reporting (CONSORT) guidelines^[38]^ and adheres to Standard Protocol Items: Recommendations for Interventional Trials (SPIRIT)^[39]^ Approvals have been granted from the Malaysian Medical Research and Ethics Committee—MREC (NMRR ID-15-851-24737) and the Monash University Human Research Ethics Committee—MUHREC (ID 9640), while the study is registered with the Australian New Zealand Clinical Trials Registry—ANZCTR (ACTRN12618000174280) with Universal Trial Number (UTN) U1111-1201-3955. The research ethics committee and trial registry are notified if there are updates or protocol amendments. The study will be administered at the Neurology Department of Hospital Kuala Lumpur (HKL) in partnership with Jeffrey Cheah School of Medicine and Health Sciences, Monash University Malaysia.

The primary aim of the study is to determine if integrating a video narrative of a doctor and a patient into the standard patient education and counseling procedure will improve medication understanding and use self-efficacy, in comparison to the current standard patient education and counseling procedure. Here, the impact of reinforcement (e.g., repetition after 3 months’ interval at T1 and T2) will also be determined. Next, the secondary aim of this study is to explore the relationship between medication self-management and stroke risk factor control, as well as its effect on the quality of life among the stroke patients. The study achieves an endpoint if stroke recurs.

### 2.2 Study population

The study will identify informed and written consented patients from an ongoing audit of all patients admitted to or seen in outpatient Neurology clinic at (HKL). HKL is the main tertiary hospital in Malaysia that receives a high number of stroke patients and referred stroke patients from various areas from Klang Valley and throughout Malaysia; approximately 1000 to 1200 acute and recurrent stroke cases annually. The targeted patients are adults (age > 18 years) of adequate literacy, diagnosed with the first stroke past 6 months, no stroke before the index event and on stroke risk preventative medications. Those patients with a diagnosis of depression, cognitive impairment or with a stroke caused by accident will not be eligible for study participation. Furthermore, only those who can read, write, and speak English or Malay language are eligible to participate in this study.

### 2.3 Sample size

A power analysis was carried out for an independent *t* test using the G∗Power version 3 to determine a sufficient sample size using an alpha of 0.05 and a power of 0.80.^[40]^ The estimation involved a medium effect size (*w* = 0.4) based on an average of effect sizes from similar recent studies associated to patient education in stroke, IMB, HBM, video narrative, and self-efficacy.^[25,41–43]^ However, sample size calculation according to stratification of stroke risk factors is not feasible because this is a disease with various underlying comorbidities and treatment heterogeneity. Thus, the desired sample size is 200 (100 each in intervention and control groups) are needed. With an estimated 15% attrition rate, 115 in each group will be recruited, thus yielding a target total sample size of 230.

### 2.4 Randomization

Randomization will be performed by using blocks of varying lengths, between 2, 4, and 6 in opaque envelopes to avoid bias. The order of the blocks and the allocations within each block will also be randomized. Assigning of patients to one of two study arms will take place after the baseline screening.

- The standard care (control group); based on MTAC framework will receive an appropriate referral for rehabilitation, nutrition counseling, and speech therapy from the neurology department. Aside from treatment review and advice, the department also provides short message service (SMS) reminders of next appointments. Pamphlets regarding common patient education information about stroke, and its preventative treatment, as well as a self-monitoring calendar, will be distributed. The information developed according to HBM constructs and MTAC guidelines is about stroke symptoms, preventative treatment adherence advice, and medication management. The patients will be provided with a general helpline in case of any inquiry pertaining to their treatment.
- The intervention group, that will receive standard care procedures, printed materials, reminders, and helpline similar to those in the control group, will also obtain a short face-to-face video narrative of a doctor and a patient's reflection about stroke.

### 2.5 The intervention

The video narrative and the video scripts have been developed from in-depth patient interviews, MTAC guidelines, HBM constructs, and stroke management guidelines. They have been designed to provide represented connected events and messages so as to motivate and to induce self-efficacy skills suited for the local context.^[15,29,44–46]^ An expert panel that consists of representatives from doctors, pharmacists, educationists, and stroke patients have employed a consensus on the video narrative and video scripts. Flesch-Kincaid reading level for the scripts, quotes, subtitles, and texts is an average grade level of six (6).^[47]^ In order to add value and to increase the impact of role model, a neurologist and a stroke patient were assigned to narrate the scripts so as to portray their true emotion and seriousness of stroke preventative measures. In addition, cues were highlighted in the video narrative as short quotes, while the subtitles were incorporated to increase comprehension of their messages.

## 3 Data collection, management, and analysis

### 3.1 Study procedures

With the supervision of a research neurologist, patient screening and randomization will be carried out by a clinic staff nurse who is not involved in this research. Patients will then complete their baseline assessments at the outpatient Neurology clinic. The neurologists will be blinded after the assignment. They will be advised to not to prompt patients regarding reception of intervention. One of the researcher (JRA) who is a pharmacist and a clinical educator, will conduct the baseline assessment. Patients randomized into the intervention arm will be displayed the video immediately after their clinic visit. All patients, regardless of study arm, will receive appropriate standard stroke treatment and medication management. At each consequent visit, the researcher will assess the level of medication understanding and use self-efficacy, while evaluation of stroke risk blood parameters will be done by the staff nurse at the clinic. The researcher will send reminder calls 3 days in advance to promote retention. At each clinic visit, both groups will receive MTAC standard care reminders to encourage self-monitoring, to modify lifestyle, to follow-up clinic appointments, and to enquire regarding their medications. Patients who miss their visits, move too far away, are referred to other outpatient clinics, and those who fail to respond up to 4 times of reminders will be noted as lost to follow-up and eventually withdrawn from the study. Also, if there is a need of unblinding due to the intervention's effect, the patient's allocation will be revealed to their neurologist and that patient is withdrawn from the study. These effects will be recorded and reported in the data analysis.

**Figure 1:**
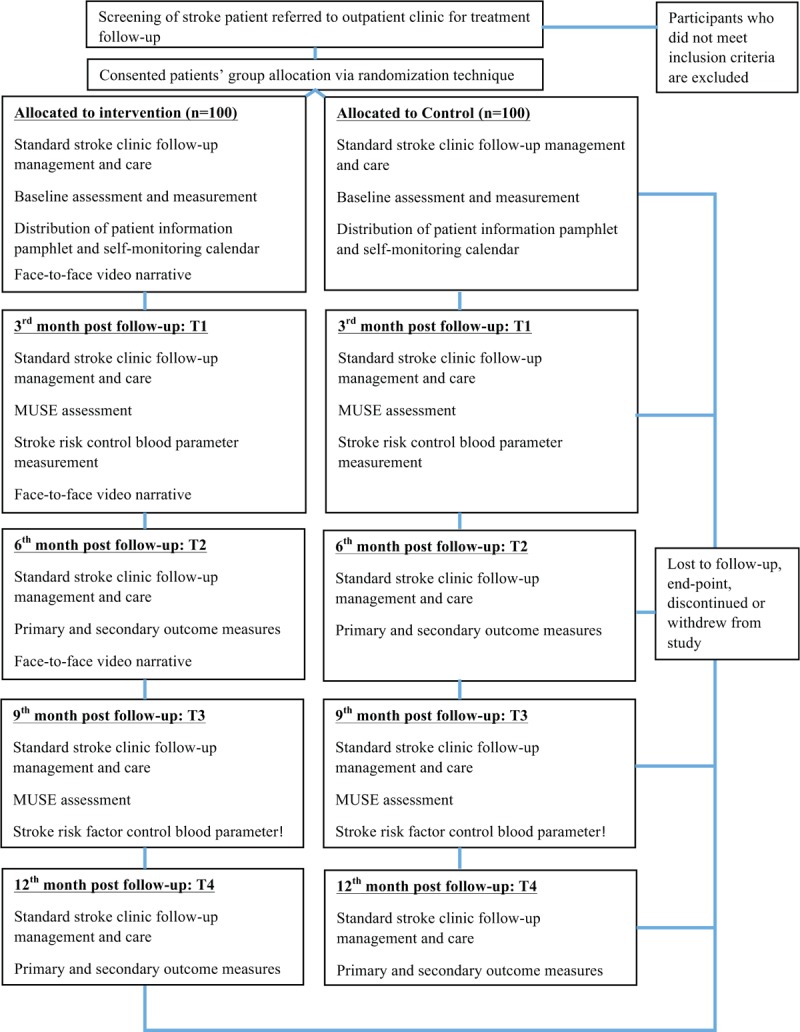
The study flow chart.

At each session, the patients will meet the neurologist first for their follow-up treatment review, and next, have further check on self-monitoring practice and lifestyle changes. The clinic staff nurse will assess their blood pressure and other appropriate blood parameters. After that, the clinic staff nurse will guide the patients into a quiet room, where the researcher will meet them individually for the outcome assessment or video viewing. The patients will view the video at baseline: T0, 3rd month: T1 and 6th month: T2 immediately after outcome measure assessment (Fig. 1).

## 4 Outcomes

### 4.1 Primary outcome measure

The primary outcome measure assessed at T0, T1, T2, T3, and T4 refers to medication understanding and use self-efficacy (MUSE).^[48]^ The study will observe the stage of medication usage behavior changes for each stroke preventative medication. MUSE with a scoring scale from 0 until 32 will be used to efficiently measure confidence in the understanding of individual's perceived ability using and adhering to the prescribed medicines, which differs from other medication-specific self-efficacy measures. Both the scales; learning and taking medication, have good internal consistency (Cronbach's alpha of 0.77 and 0.68) with acceptable construct validity and predictive validity. This scale has also been validated in the Malay language version.^[49]^

Knowledge and belief of the disease, as well as its preventative medication, have been associated with medication understanding and use self-efficacy. Therefore, the primary measures assessed at T0, T2, and T4 will also consist of validated Stroke knowledge test (SKT)^[50–52]^ and Brief illness perception questionnaire (BIPQ),^[53–55]^ whereby both have acceptable validity and reliability properties and available in the Malay language version. On top of that, this study will only adapt the BMQ specific Likert scale of the belief about medicine questionnaire^[56]^ so as to hinder redundancy and patient loading. This scale will be translated and validated in Malay language in a separate study. The SKT contains 20 questions with a scoring range of 0 to 20 regarding general information on stroke, such as pathophysiology, signs and symptoms, risk factors, and treatment methods and BIPQ, on the other hand uses a 0 to 10 scoring range of Likert-type scales for the general perception of one's illness. SKT measures differences in knowledge retention between groups whereby higher scores indicate better knowledge retention. BIPQ high scores reflect vulnerable thoughts of threatened attitudes about their disease, which will correlate to the impact of the intervention. Meanwhile, the BMQ specific is comprised of 2 scales; belief about the need of preventative medication (Necessity scale), and concerns about the potential adverse effect of the medication (Concerns scale). Higher scores indicate stronger beliefs about medicine usage.

### 4.2 Secondary outcome measures

Secondary main outcome measures include patient's stroke risk factor control which could be either on smoking habit, blood pressure, blood glucose, cholesterol, triglycerides, or international normalized ratio (INR) assessed at T0, T1, T2, T3, and T4. This outcome translates adherence to treatment and lifestyle behavioral changes such as diet and stress control, whereas assessment at T2 and T4 includes health-related quality of life, clinic appointment attendance report, and self-monitoring report.

Based on past findings, most of stroke patients were diagnosed with hypertension as their primary stroke risk factor.^[36,37]^ It is defined that blood pressure (BP) control as at or below 140/90 mmHg for patients with no diabetes and at or below 130/80 mmHg for patients with diabetes.^[45,46]^ Therefore, differences of BP will be measured at every session by using calibrated blood pressure device at the neurology clinic. Whereas, the gathered blood samples, are sent and assessed at the hospital laboratory department. The risk of recurrent stroke would be at double-fold if the patient is diagnosed with both hypertension and diabetes. Diabetes control is defined as at A1c level <6%, venous fasting plasma level <7.0 mmol/L, and random plasma level <10 mmol/L. In addition, the stroke symptoms would worsen for patients with underlying cardiovascular disease (CVD). Hence, those with hyperlipidemia control is defined as LDL-C <3.4 to 4.2 mmol/L and triglycerides <8.3 mmol/L.^[45,57,58]^ Furthermore, INR control for stroke patients with atrial fibrillation is 2.0 to 3.0.^[59]^

The overall secondary outcome measures reflect the perceived benefit to quality of life. This study will use the Short Form (36) Health Survey (SF-36) questionnaire as its quality of life outcome measure.^[60]^ The SF-36, which consist of a 36-item questionnaire, is best used in general clinical practice. The construct domains contain one's perceived physical and mental limitation, such as physical functioning, mental health, emotional problems, social life purpose, general health perceptions, and body pain. The sum of scores is between 0 (worst-perceived health state) and 100 (best-perceived health state). The measure will be able to evaluate the degree of perceived difficulty experienced between both control and intervention groups. This questionnaire has been validated in various languages with acceptable psychometric properties in many clinical settings. Besides, permission has been granted for this study to use the outcome measures from all the respective authors.

### 4.3 Statistical analysis

Data entry will be done independently by a clinic staff nurse and verified by another. Hardcopies of data collection forms and outcome measures will be kept in a locked safe, whereas softcopies are to be deposited at the repository center (Lab Archives), both at Monash University in Malaysia. Only the principal investigators (JRA), (QKF), and (TKK) have access to the interim results and makes the final decision to terminate the trial. The study team ensures that patients’ identity and personal information are coded to maintain confidentiality. In addition, (JRA) and the biostatistician (QKF), who will also be blinded, will conduct the statistical analysis by using International Business Machines Statistical Package for the Social Sciences (IBM SPSS; IBM Corp. Released 2013. IBM SPSS Statistics for Windows, Version 22.0. Armonk, NY) (v.22). The initial analysis will consist of descriptive statistics on demographic data, such as frequency distributions. Meanwhile the mean differences in primary and secondary outcomes are compared by using *t* tests or equivalent statistical method, whereas categorical outcomes are matched using chi square test. *P* values <.05 are considered statistically significant. Generalized mixed model analysis is adapted to investigate the variances between groups over time for all outcomes evaluated at the allocated time. Furthermore, the association between medication understanding and use self-efficacy, stroke knowledge, illness perception scores, as well as engagement in self-monitoring and clinic appointment attendance will be tested, as they seem to be critical indicators of behavioral changes. Multivariable analysis will be employed to examine the influence of the video narratives on stroke risk factor control and their covariates.^[61]^ Multiple imputation will be adapted to handle missing data if it occurs. In addition, withdrawn patients’ feedback about the intervention will be analyzed.

## 5 Discussion and conclusion

Persistent uncontrolled stroke risk factors can lead to unpredicted recurrent stroke event, thus making effective stroke preventive management a critical public health issue. The stroke care unit in Malaysia has limited patient education resources, especially for patients with medication understanding and use self-efficacy issues. The challenge is even higher for stroke patients who are affected by physical disability, thus hindering both motivation and self-independence. Various individuals’ illness beliefs and perceptions complicate one's thoughts towards definite health improvement and meaningful life. In such situation, the success of patient education strategies is at doubt. Patient education via video narratives has been found to be useful in primary care settings and other chronic disease clinic settings,^[32,33]^ but researches are in scarcity for effectiveness of video narrative at stroke care centers as part of the MTAC process.

As such, this study will determine if using doctor and patient video narrative could induce a positive behavioral change towards stroke risk control, whereby the initial observation should enhance medication understanding and use self-efficacy. Besides, the researcher believe that combining a doctor's perception of his stroke patients’ attitudes with a stroke patient's experiences about his or her stroke would deliver a mixed emotion of confidence and motivation towards self-efficacy. This idea, which is conceived from combined video narrative, refers to the effort to convey similar messages to stroke patients of varying learning styles and behavioral activation. This intervention is innovative as it combines health belief, motivation, and role model concept to trigger self-efficacy and self-responsibility to modify perceived negative lifestyle imbalances. Hence, it is foreseen that the discoveries from this translational research could serve as resources to further patient education development in stroke care settings. The researchers propose to share the findings to healthcare professionals and to the public via publication and conference proceedings.

## Acknowledgments

The authors would like to acknowledge the Jeffrey Cheah School of Medicine and Health Sciences, Monash University Malaysia for their financial, material, and other facilities support. The authors also wish to acknowledge the contributions of clinic staff nurses and patients from the Neurology Clinic, Hospital Kuala Lumpur.

## Author contributions

All authors have made a substantial, direct, and intellectual contribution to the work. JRA, TKK, QKF, and AZZ conceived the original concept of the study. JPJ and SSR have assisted in the study protocol development. All authors have contributed to the final design of the study protocol and have approved the final manuscript.

**Conceptualization:** Jamuna Rani Appalasamy, Kyi Kyi Tha, Kia Fatt Quek, Siva Seeta Ramaiah, Joyce Pauline Joseph, Anuar Zaini Md Zain.

**Data curation:** Jamuna Rani Appalasamy, Kia Fatt Quek.

**Formal analysis:** Jamuna Rani Appalasamy, Kia Fatt Quek.

**Funding acquisition:** Anuar Zaini Md Zain.

**Investigation:** Jamuna Rani Appalasamy, Siva Seeta Ramaiah, Joyce Pauline Joseph.

**Methodology:** Jamuna Rani Appalasamy, Kyi Kyi Tha, Kia Fatt Quek, Siva Seeta Ramaiah, Joyce Pauline Joseph, Anuar Zaini Md Zain.

**Project administration:** Jamuna Rani Appalasamy, Joyce Pauline Joseph.

**Resources:** Kyi Kyi Tha, Kia Fatt Quek, Siva Seeta Ramaiah, Anuar Zaini Md Zain.

**Supervision:** Kyi Kyi Tha, Kia Fatt Quek, Anuar Zaini Md Zain.

**Validation:** Jamuna Rani Appalasamy, Kia Fatt Quek.

**Visualization:** Jamuna Rani Appalasamy, Kyi Kyi Tha.

**Writing – original draft:** Jamuna Rani Appalasamy.

**Writing – review and editing:** Jamuna Rani Appalasamy, Kyi Kyi Tha, Kia Fatt Quek, Siva Seeta Ramaiah, Joyce Pauline Joseph, Anuar Zaini Md Zain.
